# Supplementary material for: Semi-automated quantitation of mitophagy in cells and tissues
Source: Mech Ageing Dev. 2020 Jan;185:111196. doi: 10.1016/j.mad.2019.111196 (PMC6961211; doi:10.1016/j.mad.2019.111196)
Supplement: Supplementary file 1 [file mmc1.pdf]

Semi-automated quantitation of mitophagy in cells and tissues

Lambert Montava-Garriga[1*], François Singh[1*], Graeme Ball[2*] and Ian G. Ganley[1]

[1] MRC Protein Phosphorylation and Ubiquitylation Unit, [2] Dundee Imaging Facility, School of Life Sciences University of Dundee, Dundee DD1 5EH, UK.

(*) These authors contributed equally.

Corresponding author: i.ganley@dundee.ac.uk

Running title: Mitophagy quantitation

Declarations of interest: none

Highlights:

- The *mito*-QC reporter is a powerful model to monitor mitophagy *in vitro* and *in vivo*.
- The *mito-QC Counter* is a new semi-automated tool to quantify mitophagy.
- Mitophagy is induced in ARPE19 cells and varies in distinct skeletal muscle regions.

Abstract:

Mitophagy is a natural phenomenon and entails the lysosomal degradation of mitochondria by the autophagy pathway. In recent years, the development of fluorescent pH-sensitive mitochondrial reporters has greatly facilitated the monitoring of mitophagy by distinguishing between cytosolic mitochondria or those delivered to acidic lysosomes. We recently published the *mito*-QC reporter, which consists of a mitochondrial outer membrane-localised tandem mCherry-GFP tag. This allows the quantification of mitophagy via the increase in red-only mCherry signal that arises when the GFP signal is quenched upon mitochondrial delivery to lysosomes. Here we develop a macro for FIJI, the *mito-QC Counter*, and describe its use to allow reliable and consistent semi-automated quantification of mitophagy. In this methods article we describe step-by-step how to detect and quantify mitophagy and show that mitophagy levels can be reliably calculated in different cell lines and under distinct stimuli. Finally, we show that the *mito-QC Counter* can be used to quantify mitophagy in tissues of *mito*-QC transgenic mice. We demonstrate that mitophagy levels in skeletal muscle correlates with glycolytic activity. Our present data show that the *mito-QC Counter* macro for FIJI enables the robust quantification of mitophagy both *in vitro* and *in vivo*.

Keywords: mitophagy, mitochondria, autophagy, FIJI, *mito*-QC, mitolysosome

Abbreviations: DFP, deferiprone; ROI, region of interest; stdDev, standard deviation; TEM, transition electron microscopy;

Introduction:

Mitophagy is defined as the macroautophagy of mitochondria and is a central quality control mechanism to regulate mitochondrial network homeostasis, cell survival and organism development [1–3]. Key in studying mitophagy is the ability to visualise and quantify this process. Classically, the gold standard method to investigate mitophagy (and autophagy) has been through the use of transition electron microscopy (TEM) [4]. Mitochondria within double-membraned autophagosomes can be seen at early stages of mitophagy, while at the later stages, residual fragmented mitochondria can be recognised within single-membraned autolysosomes that tend to be more electron-dense [5]. While immunogold labelling with autophagosomal and mitochondrial markers can assist in mitophagy identification, TEM itself remains a challenging and time-consuming process, especially for the inexperienced user.

Fluorescence microscopy has also been an important methodology for assessing and quantifying mitophagy. Initial hints for mitophagy were reported using mitochondrial fluorescence dyes with different sensitivity to mitochondrial membrane potential, MitoTracker Green and tetramethylrhodamine ethylester, in combination with the lysosomal dye LysoTracker Red [6]. Following on from this, the co-localisation of fluorescently labelled mitochondria with the autophagosomal marker LC3B, or endo/lysosome markers such as CD63, have been used as a readout for mitophagy [7–9]. However, given that general (non-mitophagic) autophagosomes can form at ER-mitochondria contact sites [10–12] or mitochondria themselves can directly contact lysosomes [13], the co-localisation of mitochondria with autophagosomes or lysosomes does not necessarily indicate mitophagy is occurring. To deal with this problem, the development of fluorescent reporter systems, such as *mito*-QC, mt-Keima and mito-Timer, has allowed the simple, selective and reliable assessment of mitophagy *in vitro* and *in vivo* and this has revolutionised the field [14–18].

The *mito*-QC reporter consists of a fluorescent pH-biosensor system of a tandem mCherry-GFP tag attached to an outer mitochondrial membrane localisation signal derived from the

mitochondrial protein FIS1 (amino acids 101-152) [14,15]. This assay exploits the acid-labile properties of GFP and mCherry to monitor mitophagy, as while GFP fluorescence becomes quenched in the lysosome, the mCherry signal does not [4,15]. Hence, mitophagy can be quantified by the increase in the number and size of mCherry-only puncta that corresponds to mitochondria delivered to lysosomes. The specificity of the *mito*-QC reporter for mitophagy was recently confirmed with an *in vivo* side-by-side comparison with an almost identical reporter that can assess autophagy in general [19]. The mt-Keima fluorescent probe system uses a pH-sensitive coral-derived protein with a pH-dependent shift in fluorescence excitation to assess mitophagy in cells and tissues [16,17]. Thus, mt-Keima has a single emission peak at 620 nm and two excitation peaks at 543 nm (ionised state at low pH) and 458 nm (neutral state at high pH), where the degree of mitophagy is assessed with the ratio between both excitation peaks (543/458 nm) [16]. Lastly, the mito-Timer reporter relies on the fluorescence shift of DsRed1-E5 fluorophore from green to red over time as the protein matures [20]. Once combined with LAMP1 staining, one can assess mitophagy [21]; otherwise, it provides an indirect measurement. However, it is a powerful tool to monitor the age and biogenesis of mitochondria [18]. Notably, besides microscopy, the changes in fluorescence of these reporters upon mitophagy induction can also be quantified using flow cytometry [18,22,23].

Mitophagy can also be measured using biochemical approaches that rely on measuring loss of mitochondrial mass. Different methodologies have been implemented over the years, which involve the quantification of a decrease in mitochondrial markers including mitochondrial DNA content, citrate synthase activity or immunoblotting of mitochondrial proteins [15,22,24,25]. However, the use of inhibitors of lysosome activity (e.g. Bafilomycin A1 or E64d and Pepstatin A) are required to confirm the lysosomal-dependent degradation of mitochondria [4,15]. These methodologies have been more commonly used in the context of overexpressed Parkin-dependent mitophagy, where prolonged periods of mitochondria depolarization lead to an almost total loss of mitochondrial proteins [9]. However, these

approaches are less sensitive to assess endogenous mitophagy compared to the aforementioned fluorescence reporters. They require a significant loss in mitochondrial mass to confidently quantify mitophagy, which can differ between cell types and mitophagy stimuli. For a more in-depth comparison of the available methods to monitor mitophagy *in vitro* and *in vivo,* we refer the reader to a recent methods article [26].

In this methods article, we focused on the assessment of mitophagy using the *mito*-QC reporter assay, though our developed system is applicable to any dual-colour reporter system, such as the previously mentioned mt-Keima. The increase in the number of mCherry-only fluorescing puncta (which represent mitolysosomes (lysosomes containing mitochondria)) per cell or field has been the conventional read-out to quantify mitophagy. To determine the number of positive mitolysosomes, quantification has previously been undertaken either manually or using specialised image analysis software, which primarily involves license-based platforms such as Volocity (Quorum Technologies) or Imaris (Oxford Instruments) [14,15,27,28]. While both approaches have been successfully used to assess mitophagy, they also have caveats. For example, manual counting can lead to subjectivity in the criteria used to define a positive mitolysosome and thus variation between different researchers and laboratories. It is also not time-effective or scalable for experiments involving large volumes of data. Volocity and Imaris offer a robust and reliable platform to perform a semi-automatic and multiparametric analysis of mitophagy, but they require expensive licences that can limit the accessibility to a broader scientific community. Given the pervasive use of FIJI (ImageJ; NIH) as a freeware platform for image analysis, we developed a macro for FIJI, termed "*mito-QC Counter",* which will be described here together with the methodology to reliably quantify mitophagy using the *mito*-QC reporter assay.

The *mito-QC Counter*: a new image analysis tool to assess mitophagy

The *mito-QC Counter* is a semi-automatic, user-friendly and cost-effective macro for FIJI. The underlying principle to detect and quantify mitophagy relies on the difference in fluorescence intensity between mCherry and GFP proteins that occurs when the *mito*-QC reporter is delivered to lysosomes. In normal conditions outside the lysosome, the pixel intensities of GFP and mCherry from the *mito*-QC reporter should be similar and the ratio of mCherry/GFP intensities for each of these pixels should be close to 1, using typical imaging parameters. However, the quenching of GFP fluorescence in the lysosomes will result in pixels with reduced GFP intensity relative to mCherry. Pixels within mitolysosomes will therefore display a higher ratio value of mCherry/GFP intensity than the rest of the pixels with reporter fluorescence across the cell. Hence, to detect mitophagy, the macro creates a new image by dividing the intensity of each pixel in the mCherry channel by the equivalent in the GFP channel from the source image. The resulting ratio image enables us to highlight the "red dots", or mitolysosomes, which appear as high mCherry/GFP intensity ratio puncta. The macro detects peaks in this ratio image and filters these using a minimum red channel intensity threshold to exclude spurious high-ratio pixels that are typically found in low-intensity regions (e.g. pixels in background regions that happen to have a high red/green ratio due to noise). In a similar way, objects can be detected by thresholding the ratio image to generate a binary mask and excluding pixels where the red channel intensity is below a threshold value. These peaks and object pixels are then quantified.

The *mito-QC Counter* workflow is schematically depicted in Figure 1A, which is comprised of four key steps (Figure 1A and B). Step 1 involves the identification of cells or regions of tissue to quantify, which have to be manually defined and saved as regions of interest (ROI) for each image. Between Step 1 and 2, upon starting the *mito-QC Counter*, a command window appears in which the user must assign the mCherry and GFP channels to their corresponding number in the source image (e.g. Channel 1 or Channel 2). Before the analysis, images are first filtered to reduce the background noise with a median filter. The

pixel radius to define the size of the filtering matrix can be introduced at this point, where a higher radius will result in higher removal of image noise but can also lead to excessive blurring. The two thresholds used for mitolysosome number quantification are also defined at this stage (Figure 1A). The first threshold is the mCherry/GFP intensity ratio threshold. Local intensity variations in background pixels can often lead to ratio values greater than the set threshold, which would be detected as false positives. To prevent this, a second threshold is applied using the intensity of the mCherry channel. Only peaks/pixels with an above-threshold mCherry intensity will be counted. To set the mCherry intensity threshold we use a strategy of automatically calculating the mean mCherry intensity and standard deviation of intensities for each image and allowing the user to define how many standard deviations (stdDev) from the mean (either positive or negative) should be used for this threshold. Furthermore, a “batch” processing mode can be selected to process all the images with pre-selected ROIs saved in a defined folder; facilitating rapid analysis of multiple images.

In Step 3, the macro uses the “Find Maxima” function of FIJI to detect high intensity peaks in the ratio image (Figure 1B). Then, in Step 4, an mCherry intensity threshold is applied to exclude peaks in background regions (Figure 1B). The macro generates a black and white mask image that we term a “mitophagy mask”, from the mCherry/GFP ratio image and the thresholding criteria. This mask image provides key information to determine the total area and mean size of mitolysosomes as well as enabling visualisation of the quantified mitophagy areas (Figure 1A and 1B). Finally, the macro produces a table containing the measurements and parameters described in Table 1 for each cell ROI quantified. In batch mode, the *mito-QC Counter* automatically exports the data in a comma-separated value (.csv - file readable by most spreadsheet software), while it saves the mitophagy mask image, mCherry/GFP ratio image and an image with the detected mitolysosomes plus original cell ROIs in a new folder. It should be noted that the quantification of mitolysosomes derives from the mCherry/GFP intensity ratio image, while the values for mitolysosome areas derive from the mitophagy mask.

Protocol on how to use the *mito-QC Counter*:

A. Download and install the *mito-QC Counter* macro

1. The file “***mQC_counter.ijm”*** ("version 1.0", DOI: 10.5281/zenodo.3466642) is available at the following address: https://github.com/graemeball/mQC_counter

2. Paste this file in the following folder:

   Windows:

   (the folder where you saved FIJI)\fiji-win64\Fiji.app\plugins\Scripts\Plugins

   Mac:

   Right click on the FIJI program and select “show package contents”.

   Select plugins→Scripts>Plugins and paste the file in this subfolder.

3. Start FIJI and install the plugin

   →Plugins→Macros→Install…

   Restart FIJI

4. We advise creating a shortcut for the plugin

   Plugins→Shortcut→Add Shortcut…

   (Example: F6)

   Restart FIJI

B. Find the appropriate settings for *batch* analysis using *the single image mode*

1. Open a picture with high mitophagy (e.g. cells stimulated with deferiprone for 24 h [15])

   Make sure the following options are selected in the *Bio-Formats Import Options* window that appears:

   View stack with: Hyperstack

   Color mode: Composite

   Click OK

2. Adjust the green channel brightness

   →Image→Adjust→Brightness/contrast...

A new window appears called B&C.

Slide the maximum cursor to the left until you can visualise the cell borders. This is needed so that the cell outline can be noted in the next step.

3. Select the "***Freehand Selection***" tool (Bean-shaped selection tool icon, 4th from left)

   Draw around the first cell, then press "**t**"

   Repeat with all cells in the picture.

4. Start the macro (F6).

   A new dialog widow will appear. Make sure your green and red channels correspond to the values shown. Do not tick the batch box.

   In general, a radius for smoothing value of 1 is sufficient and there should be no need to change this value unless the image is oversampled or very noisy.

   Adjust the *mito-QC Counter* parameters: start with the default settings and press **OK**

   If results are not satisfactory using default threshold parameters, repeat this step and change the thresholds to exclude false negatives (missing peaks) and false positives (usually in low-intensity background regions).

   See troubleshooting Table 2 for more information.

   Once you have found satisfactory parameters, check that they are appropriate with images of different treatments/genotypes.

C. Batch processing with the *mito-QC Counter*

1. Open the first picture

   Outline the first cell as above, then press "**CTRL"+"B**" to add the selection to the image overlay.

   Repeat with all cells in the picture.

   Press **Reset** in the B&C window

   Save the picture as a Tiff in a new folder (make 1 folder per condition)

   For the first picture: →File→Save as→Tiff…

   For the next pictures: "**CTRL"+"S**"

Repeat for all images.

2. Start the plugin (F6).

   A new window appears, tick the batch box and use the parameters previously determined. Press **OK**, a new window will appear and you can now select the folder you have created containing the Tiff files with overlays.

   The plugin will automatically save for each image in that same folder:

   - a .csv file with all your results (see Table 1).
   - a mCherry/GFP ratio image.
   - a image with an overlaid of cell ROIs and detected peaks.
   - a “mitophagy mask” image which enables the visualisation of mitophagy.

Implementing the *mito-QC Counter* to study mitophagy *in vitro*:

To evaluate how the *mito-QC Counter* quantifies mitolysosomes and assesses endogenous mitophagy, we first focused on mitophagy induced *in vitro*. We previously showed that the neuroblastoma cell line derived from bone marrow, SH-SY5Y, undergoes mitophagy under different stimuli, with iron chelation using deferiprone (DFP) being the most potent inducer [15]. In parallel, we also tested a different cell line, ARPE-19, which are spontaneously immortalised and non-transformed cells derived from human retinal pigmented epithelium [29]. We compared DFP-induced mitophagy in SH-SY5Y and ARPE-19 cells using our new macro (Figure 2A and B). Consistent with previous results [15], the *mito-QC Counter* could successfully report an increase in the total number of mitolysosomes in SH-SY5Y cells treated with DFP (Figure 2A and C) [15]. ARPE-19 cells also showed a strong response to DFP, which resulted in a robust increase in the total number of mitolysosomes (Figure 2B and C). Interestingly, the total number of formed mitolysosomes in ARPE-19 cells was approximately 3 times higher than SH-SY5Y cells (Figure 2C), thus proving the macro’s sensitivity to detect different levels of mitolysosomes between different cell types. However, ARPE-19 cells are larger than SH-SY5Y cells, and as a result, the proportion of formed

mitolysosomes between both cell type after DFP stimulation was in fact similar once the cellular area is taken into account (Figure 2D). Consistently, the percentage of total mitolysosome area quantified from the mitophagy mask showed a similar increase between SH-SY5Y and ARPE-19 cells (Figure 2E). The *mito-QC Counter* also provides information on the mean size of mitolysosomes detected in the mitophagy mask, which was significantly increased with DFP treatment in both cell lines but was slightly higher in ARPE-19 cells (Figure 2F). It is worth noting that the size of mitolysosomes can provide valuable information about mitophagy induction and autophagosome-lysosome trafficking. Multiple mitophagosomes (autophagosomes containing mitochondria) can fuse with the same lysosome, while multiple mitolysosomes can also fuse together. Therefore, the induction of mitophagy may also involve an increase in mitolysosome size, which may not be directly reflected in the total number of red-only dots. Besides, this measurement could also provide relevant information on defects in lysosome trafficking and fusion due to a specific treatment or phenotype, which could result in smaller or larger mean of mitolysosomes size.

We next sought to further test the macro's performance by comparing the number of formed mitolysosomes in SH-SY5Y cells treated with DFP through manual counting or using the *mito-QC Counter* with different threshold settings (Figure 3). The mCherry/GFP ratio threshold for maximum local intensity is central to accurately determine positive mitolysosomes within ROIs. Hence, we tested four different ratio thresholds and showed that a threshold of 0.6 yielded a similar quantification of mitolysosomes as the manual assessment (Figure 3A). While the macro's default threshold of 0.5 also showed a similar result to manual counting, relaxing the threshold to 0.4 or 0.2 resulted in a substantial increase in the number of quantified mitolysosomes, which resulted from the detection of background noise (Figure 3A). In parallel, we also examined how the radius for smoothing the image can impact the quantification (Figure 3B). Using an mCherry/GFP ratio threshold of 0.5 for example, the quantification of unfiltered images (Radius=0) resulted in an increased number of counted mitolysosomes, while an excessive filtering with high values of ratio (e.g.

2 or 3) resulted in a substantial loss of accuracy in the *mito-QC Counter* quantification relative to manual counting. A radius value of 1 proved to be the optimal choice in most cases (Figure 3B).

Lastly, we tested the impact of the mCherry intensity threshold using a constant value of mCherry/GFP ratio threshold (0.5) and smoothing radius (1) (Figure 3C). As expected, an increase to +1, +2 or +3 stdDev above the mean mCherry intensity resulted in a more stringent threshold and a corresponding decrease in the number of detected mitolysosomes (Figure 3C). In contrast, negative standard deviations below the mCherry mean intensity did not yield changes in the quantification relative to 0 stdDev (Figure 3C), indicating that candidate mitolysosomes within the ROIs were already above this lower mCherry intensity threshold. Interestingly, the use of +1 stdDev above the mean reduced the number of mitolysosomes counted using a 0.5 ratio threshold to a level similar to that obtained by manual counting, which may indicate that some of the mitolysosomes detected are mCherry-intensity peaks close to the mean value. Notably, the main function of the mCherry intensity threshold is to prevent the detection of false-positive mitolysosome counts in background regions.

Taken together, the benchmarking of different thresholds highlighted that optimisation of the thresholding settings for the *mito-QC Counter* is crucial to avoid the under detection of mitolysosomes or an increased detection of false-positive. The thresholds default values are 0.5 for the Ratio threshold and 0 for the radius for smoothing the image, but they should be adjusted on a case-by-case basis for each experiment and the microscope used. Furthermore, it is important to note that the same microscope and image acquisition parameters must be used within each experiment to achieve reliable and comparable quantifications. Ultimately, to confirm that the *mito-QC Counter* was indeed quantifying mitolysosomes, we showed that the red-only structures, formed under DFP stimulation and counted by the *mito-QC Counter,* co-localised with the lysosomal marker LAMP1 (Figure 4).

To further confirm the versatility and reliability of the *mito-QC Counter*, we used the macro to quantify mitophagy induced by hypoxia in ARPE-19 cells treated with or without bafilomycin A1 (Figure 5A). In the presence of bafilomycin A1, the alkalization of the lysosome elicits dequenching of the GFP signal within mitolysosomes, which would then no longer be observed as red-only puncta and therefore not quantified by the macro. Hypoxia triggered an increase in the number of mitolysosomes per cell or cells' area (Figure 5B and C), which was not as robust as DFP (Figure 2C and D). The percentage of cell's area corresponding to mitolysosomes was also increased with hypoxia (Figure 5D), while the macro showed that the mean size of formed mitolysosomes in hypoxia was approximately half than DFP (Figure 5E and 2F). Importantly, cells subjected to hypoxia in the presence of bafilomycin A1 showed a significant reduction in the detection of mitolysosomes by the *mito-QC Counter* (Figure 5A-C). Consistently, the macro also reported a reduction in the percentage of mitolysosome area and a reduction in the mitophagosome size in the presence of bafilomycin A1 (Figure 5D and E). Thus, this result demonstrated the specificity and robustness of our macro for detecting genuine mitolysosomes from the *mito*-QC reporter assay.

Therefore, we confirmed here that the *mito-QC Counter* is a specific, sensitive and reliable tool to quantify meaningful changes in mitophagy using the *mito*-QC reporter, which included different cell types and stimuli. To the best of our knowledge, we provided here the first observations showing how ARPE-19 cells undergo mitophagy under DFP and hypoxia; indicating that this cell line can be used as an *in vitro* model to study stress-induced mitophagy.

Quantifying mitophagy *in vivo* using the *mito-QC Counter:*

Next, we assessed the efficacy of our macro in mouse tissue sections. To demonstrate its versatility, we chose to evaluate the macro in a highly heterogenous tissue in terms of mitochondrial content: the skeletal muscle. Hindlimb muscles are characterised by a

metabolic gradient, with a lateral portion composed by a highly glycolytic muscle (white gastrocnemius), and a medial part composed by a highly oxidative one (soleus). In between these two muscles, the gastrocnemius can be further subdivided in two other heterogenous regions: the red gastrocnemius, which is mostly oxidative, and the mixed gastrocnemius, which is mostly glycolytic and contains few oxidative fibres. In addition to a difference in mitochondrial content, metabolically distinct muscles have been reported to have different antioxidant defences, ROS production rates, and resistance to apoptotic stimuli [30,31]. However, the basal rates of mitophagy in skeletal muscles are still poorly understood. We observed in our *mito*-QC reporter mice, that the expression of the reporter was proportional to the oxidative metabolism of the muscle fibres (Figure 6A and B). Hence, this feature enables the visualisation of the metabolic gradient occurring in the hindlimb, and an easy delineation of our four regions of interest. Although red-only dots are difficult to visualise in the composite images, the *mito-QC Counter* enables their visualisation as a separate black and white channel (Figure 6C). We observed an inverse correlation between metabolism and mitophagy. Indeed, the highly oxidative soleus displayed a diminished number of mitolysosomes per tissue area compared to glycolytic muscles (Figure 6D), as well as a lower proportion of area occupied by mitolysosomes (Figure 6E). However, it is interesting to note that the mean mitolysosome size was higher in the soleus than in the other muscles (Figure 6F). This observation in highly oxidative fibres can be explained by the presence of enlarged mitolysosomes within subsarcolemmal mitochondria. The proportional expression of the *mito*-QC reporter relative to metabolic activity enabled us to extrapolate the intensity of the reporter (in the green channel) per area of tissue as a readout for mitochondrial content (Figure 6G). Interestingly, when expressing the mitolysosome number and area as a function of this estimate for mitochondrial content (i.e. per mitochondrial unit), the differences between the muscles become even more striking (Figure 6H-I).

Our observations confirm that the *mito-QC Counter* (using the same parameters for each region) is suitable for the detection of mitophagy in heterogenous tissues. Our findings also

underline a clear inverse relationship between metabolism and mitophagy in skeletal muscles.

Conclusion:

The development of pH-sensitive fluorescence reporters such as the *mito*-QC reporter has significantly expanded our possibilities to study mitophagy *in vitro* and *in vivo*. However, a limiting factor to completely unleash the potential of the *mito*-QC reporter has been the access to reliable quantification methods, which is critical to assess the number, size or shape of mitolysosomes. Thus, we developed and implemented here the *mito-QC Counter,* a new open source macro for FIJI (ImageJ) that facilitates a time-effective, cost-effective and reliable strategy to assess mitophagy using the *mito*-QC reporter. Though we have focussed on quantifying mitophagy in fixed samples in this instance, *mito-QC Counter* can also be used to analyse live samples through the quantitation of individual frames from time-lapse experiments.

Here, we provided a detailed protocol on how to use the macro, while we demonstrated that the *mito-QC Counter* could faithfully quantify changes in mitophagy *in vitro* and *in vivo*, as previously observed [15,28]. Furthermore, we identified that ARPE-19 cells undergo stress-induced mitophagy, which can thus be used as a new cell-line model to study mitophagy. In parallel, the implementation of the *mito-QC Counter in vivo* provided further evidence on the important interlink between tissues' metabolic context and basal mitophagy, which is still mostly unexplored and vital to fully understand how mitophagy is regulated *in vivo*.

In conclusion, the *mito-QC Counter* can be a powerful tool for the scientific community to assess mitophagy with the *mito*-QC reporter *in vitro* and *in vivo.* Nonetheless, we envisage that the utility of the *mito-QC Counter* can be easily expanded to other systems using the *mito*-QC reporter's dual colour idiosyncratic properties, such as mt-Keima, as well for reporters of other organelles/proteins of interest. Thus, the *mito-QC Counter* could also facilitate the assessment of other selective autophagy pathways beyond mitophagy.

Column title	Measurement
Image	Analysed image name.
CellROI	Region of interest within the image.
CellArea_µm2	Area of the ROI.
greenMean	Mean of green intensity within the ROI.<br>This value can be extrapolated as a reading for mitochondrial content.
redMean	Mean of red intensity within the ROI.
RGratio	= redTotal/greenTotal
nMitolysosomes	Number of mitolysosomes detected within each ROI/cell.
nML_per_um2	Mitolysosomes/cell area<br>= nMitolysosomes/CellArea_µm^2
nML_over_green	Mitolysosome/mitochondrial content<br>= nMitolysosomes/greenMean
MLtotalArea_µm2	Total area of the mitolysosomes within the ROI.
MLpctArea	Percentage of the cell occupied by mitolysosomes.<br>= (mitoArea_µm^2/ CellArea_µm^2)*100
MLmeanArea_µm2	Mean mitolysosome size<br>= mitoArea_µm^2/ nMitolysosomes
MLarea_µm2_over_green	Mitolysosome area/mitochondrial content<br>= MLtotalArea_µm^2/greenMean
X_tl	X coordinate of the ROI bounding rectangle top left corner.
Y_tl	Y coordinate of the ROI bounding rectangle top left corner.
ratioThresh	Threshold used for detecting the peaks of intensity in the ratio channel.<br>User-defined in the settings of the *mito-QC Counter*.
redThresh	Threshold used for the red channel.<br>User-defined in the settings of the *mito-QC Counter*.<br>By default, this value is the mean red intensity of the image.
smoothRad	Radius for smoothing.<br>User-defined in the settings of the *mito-QC Counter.*

Table 1: Measurements made and parameters used by the mito-QC Counter

Problem	Solution
Not enough dots detected.	Decrease the Ratio image threshold. (ex:0.4)
	Decrease the spot red intensity threshold. (ex: mean(0) -1 stDev).
Too many dots detected.	Increase the Ratio image threshold (ex:0.6)
	Increase the spot red intensity threshold (ex: mean(0) +1 stDev).
Unspecific dots (especially at the border of your cell).	Increase the spot red intensity threshold (ex: mean(0) +1 stDev).
Picture is very noisy.	Increase the radius for smoothing
High background fluorescence can lead to excessive detection of mitolysosome area and unreliable counting.	Optimise experimental conditions to reduce background.<br>We advise avoiding the use of mounting media containing nuclear dyes.<br>Instead, counterstaining with nuclear dyes is possible if performed before the mounting step.
Heterogenous population of cells expressing the reporter (viral or transient transfection).	Estimation of mitochondrial content and linked parameters cannot be used in these conditions.<br>If these parameters need to be assessed, we advise FACS sorting the cells to obtain a homogenous population.
Bleaching of mCherry and/or GFP signal can lead to different ratios between pictures of the same sample and aberrant results in the mitophagy mask.	Optimise sample preparation and/or acquisition parameters to reduce bleaching.

Table 2: Troubleshooting tips to use the mito-QC Counter

Material and Methods:

Assessment of mitophagy in cell lines *in vitro*:

Experiments were conducted in either SH-SY5Y or ARPE-19 cells following American Type Culture collection (ATCC) guidance. In brief, cells were cultured in 1:1 DMEM:F-12 media (Thermo Scientific; #12634010) supplemented with 10% (v/v) FBS (Sigma; #F7524), 2 mM L-glutamine (Thermo Scientific; #25030-024), 100 U/ml penicillin and 0.1 mg/ml streptomycin (Thermo Scientific; #15140-122) and incubated at 37 °C with 5% CO_2 in a water-saturated incubator. The *mito*-QC reporter was retrovirally transfected as previously described [15]. Mitophagy was assessed from pools of cells transfected with the reporter. Iron chelation induced mitophagy with Deferiprone (DFP) (Sigma; #379409) was achieved as previously

reported, where cells were treated with 1 mM DFP, dissolved in water before use with a pulse of heat (90 °C), for 24 hours. For hypoxia treatment cells were transferred to a Ruskinn INVIVO2 300 workstation (Ruskinn/Baker) at 37 °C in 1% O_2 and 5% CO_2, where were incubated for 42 hours. After 36 hours incubation in hypoxia, 50 nM of Bafilomycin A1 (Enzo; BML-CM110) was added to inhibit lysosome acidification during the remaining 6 hours up to 42 hours.

The experiments using the *mito*-QC reporter to assess mitophagy were processed as previously described [15]. In brief, cells containing the *mito*-QC reporter were washed once with PBS (Thermo Scientific; #14190-094) and fixed with 3.7% PFA (Sigma; P6148) in 200 mM HEPES (Formedium; HEPES10), pH 7.0 for 10 minutes at room temperature. Then, samples were washed twice with DMEM media supplemented with 10 mM HEPES pH 7.0 and 0.04% Sodium Azide (Sigma; S8032), followed by a 10 min incubation. After two washes with PBS, coverslips were incubated with 1 µg/ml Hoechst (Thermo Scientific; #62249) for 30 minutes, washed three times with PBS, dipped in MilliQ water and mounted onto glass slides with ProLong Diamond Antifade Mountant (Thermo Scientific; #P36961).

The immunostaining of SH-SY5Y *mito*-QC reporter cells with LAMP1 was performed as follow: cells were washing once with PBS and fixing cells with 3.7 % PFA pH 7.0 for 20 minutes at room temperature. The cross-linking reaction was quenched as described above. Cells were the permeabilised with 1% BSA (Roche; #10735108001)/PBS + 0.1% NP-40 (Merck/Millipore; #492016) for 3 minutes at room temperature. In contrast to tissue preparations, the permeabilization of cell *in vitro* can affect the red-only mitolysosome stability. Therefore, the quantification of mitophagy using the *mito*-QC reporter should be avoided after detergent permeabilization of cells in vitro. Following permeabilization and fixation, coverslips were next washed two times with 1% BSA/PBS and followed by a 30 minutes incubation at room temperature. The LAMP1 primary antibody (Santa Cruz; sc-20011) were incubated at 1/300 in 1% BSA/PBS for 1.5 hours at 37 °C. Then, coverslips

were washed with 1% BSA/PBS four times of 10 minutes each. The secondary anti-mouse Alexa Fluor 647 nm antibody (Thermo Scientific; #A21236) was incubated at 1/1000 in 1% BSA/PBS for 30 minutes in the dark at room temperature. Coverslips were washed again four times with 1% BSA/PBS for 10 minutes each. Cells were then stained with Hoechst and mounted glass slides as described above.

Images with the *mito*-QC reporter to quantify mitophagy were acquired using a Nikon Eclipse Ti widefield microscope (Plan Apo Lambda 60x Oil Ph3 DM) with the NIS-Elements software, while the images for the LAMP1 co-localisation experiment were acquired in a Leica SP8 laser scanning confocal microscope (HC PL APO 63x/1.40 oil CS2). All the images were processed with FIJI v1.52n software (ImageJ, NIH). Quantification of mitophagy was performed from three independent experiments counting over 50 cells for condition. Images were processed with the *mito-QC Counter*. For images acquired in the widefield microscope the following parameters were used: Radius for smoothing images = 1, Ratio threshold = 0.6, and Red channel threshold = mean + 0 standard deviation. For images acquired in the Leica SP8 confocal microscope the following parameters were used: Radius for smoothing images = 2, Ratio threshold = 1.5, and Red channel threshold = mean + 0.5 standard deviation.

Assessment of mitophagy in mouse tissues *in vivo*:

Experiments were performed on *mito*-QC (mCherry-GFP-Fis1[101-152]) reporter mice that were generated as previously described [14,28]. Experiments were performed on 4 adult mice (17-19 weeks old) of both genders, all homozygous for the *mito*-QC reporter. Animals were housed in sex-matched littermate groups of between two and five animals per cage in neutral temperature environment (21° ± 1 °C), with a relative humidity of 55-65%, on a 12:12 hour photoperiod, and were provided food and water *ad libitum*. All experiments were performed in agreement with the guidelines from Directive 2010/63/EU of the European Parliament on the protection of animals used for scientific purposes. All animal studies and breeding were approved by the University of Dundee ethical review committee, and further subjected to

approved study plans by the Named Veterinary Surgeon and Compliance Officer (Dr. Ngaire Dennison) and performed under a UK Home Office project license in agreement with the Animal Scientific Procedures Act (ASPA, 1986).

Mice were terminally anesthetised with an intraperitoneal injection of pentobarbital sodium (Euthatal, Merial) then trans-cardially perfused with Dulbecco's PBS (DPBS: Gibco, 14190-094) to remove blood. Tissues were collected and processed by overnight immersion fixation in freshly prepared 3.7% Paraformaldehyde (Sigma, P6148), 200 mM HEPES, pH=7.00. The next day, fixed tissues were washed three times in DPBS, and immersed in a sucrose 30% (w/v) solution containing 0.04% sodium azide. Samples were stored at 4ºC in that sucrose solution until further utilisation.

Tissues were embedded in an O.C.T. matrix (Scigen, 4586) and frozen-sectioned with a cryostat (Leica CM1860UV). 12 microns sections were placed on slides (Leica Surgipath® X-tra™ Adhesive, 3800202), and then air dried and kept at -80ºC until further utilisation. Sections were thawed at room temperature and washed 3 times 5 minutes in DPBS (Gibco, 14190-094). Slides were then mounted using Vectashield Antifade Mounting Medium (Vector Laboratories, H-1000) and high precision cover glasses (No. 1.5H, Marienfeld, 0107222) and sealed with nail polish.

Confocal pictures were obtained by uniform random sampling using a Zeiss 710 laser scanning confocal microscope (Plan-Apochromat 63x/1.4 Oil DIC M27) using the optimal parameters for acquisition (Nyquist). Representative tile scan was obtained using a 10x objective (EC Plan-Neofluar 10x/0.3). Representative images intensities were enhanced to improve visualisation in figures. Quantification of mitophagy was realised on 6 pictures per sample. Images were processed with the *mito*-QC Counter with the following parameters: Radius for smoothing images=1, Ratio threshold=1, and Red channel threshold=mean+1 standard deviation.

Statistics:

Data are represented as either boxplot with Tukey whiskers with outliers depicted as individual dots or scatter bar plots with each data point representing the mean value of an individual experiment or animal subject ± standard deviation (SD) and the bar the total mean. Numbers of experiments or subjects are indicated in the respective figure legends. All statistical analyses were performed in GraphPad Prism version 8.2.1 (www.graphpad.com). Student's t-test was used for pairwise comparisons, whereas multiple comparisons were analyzed with either one-way or two-way analysis of variance (ANOVA) and either Tukey's or Bonferonni's multiple comparison post-test as indicated in the figure legends. Statistical significance is displayed as *p<0.05: **p<0.01, ***p<0.001, ****p<0.0001.

Acknowledgements:

We would like to thank members of the Ganley Lab for trialling different versions of the *mito-QC Counter* and providing feedback. This work was funded by a grant from the Medical Research Council, UK (IGG; MC_UU_00018/2).

References:

[1] L. Montava-Garriga, I.G. Ganley, Outstanding Questions in Mitophagy: What we Do and Do Not Know, J. Mol. Biol. (2019). doi:10.1016/j.jmb.2019.06.032.

[2] F. Suomi, T.G. McWilliams, Autophagy in the mammalian nervous system: a primer for neuroscientists, Neuronal Signal. 3 (2019) NS20180134. doi:10.1042/NS20180134.

[3] S. Pickles, P. Vigié, R.J. Youle, Mitophagy and Quality Control Mechanisms in Mitochondrial Maintenance, Curr. Biol. 28 (2018) R142–R143. doi:10.1016/j.cub.2018.01.004.

[4] N. Mizushima, T. Yoshimori, B. Levine, Methods in Mammalian Autophagy Research,

Cell. 140 (2010) 313–326. doi:10.1016/j.cell.2010.01.028.

[5] E.-L. Eskelinen, F. Reggiori, M. Baba, A.L. Kovács, P.O. Seglen, Seeing is believing: The impact of electron microscopy on autophagy research, Autophagy. 7 (2011) 935–956. doi:10.4161/auto.7.9.15760.

[6] S.P. Elmore, T. Qian, S.F. Grissom, J.J. Lemasters, The mitochondrial permeability transition initiates autophagy in rat hepatocytes., FASEB J. 15 (2001) 2286–7. doi:10.1096/fj.01-0206fje.

[7] E.T.W. Bampton, C.G. Goemans, D. Niranjan, N. Mizushima, A.M. Tolkovsky, The Dynamics of Autophagy Visualised in Live Cells: from Autophagosome Formation to Fusion with Endo/lysosomes, Autophagy. 1 (2005) 23–36. doi:10.4161/auto.1.1.1495.

[8] I. Kim, J.J. Lemasters, Mitochondrial degradation by autophagy (mitophagy) in GFP-LC3 transgenic hepatocytes during nutrient deprivation, Am. J. Physiol. Physiol. 300 (2011) C308–C317. doi:10.1152/ajpcell.00056.2010.

[9] D. Narendra, A. Tanaka, D.F. Suen, R.J. Youle, Parkin is recruited selectively to impaired mitochondria and promotes their autophagy, J. Cell Biol. 183 (2008) 795–803. doi:10.1083/jcb.200809125.

[10] J. Biazik, P. Ylä-Anttila, H. Vihinen, E. Jokitalo, E.-L. Eskelinen, Ultrastructural relationship of the phagophore with surrounding organelles, Autophagy. 11 (2015) 439–451. doi:10.1080/15548627.2015.1017178.

[11] M. Hamasaki, N. Furuta, A. Matsuda, A. Nezu, A. Yamamoto, N. Fujita, H. Oomori, T. Noda, T. Haraguchi, Y. Hiraoka, A. Amano, T. Yoshimori, Autophagosomes form at ER–mitochondria contact sites, Nature. 495 (2013) 389–393. doi:10.1038/nature11910.

[12] D.W. Hailey, A.S. Rambold, P. Satpute-Krishnan, K. Mitra, R. Sougrat, P.K. Kim, J. Lippincott-Schwartz, Mitochondria Supply Membranes for Autophagosome Biogenesis

during Starvation, Cell. 141 (2010) 656–667. doi:10.1016/j.cell.2010.04.009.

[13] Y.C. Wong, D. Ysselstein, D. Krainc, Mitochondria–lysosome contacts regulate mitochondrial fission via RAB7 GTP hydrolysis, Nature. 554 (2018) 382–386. doi:10.1038/nature25486.

[14] T.G. McWilliams, A.R. Prescott, G.F.G. Allen, J. Tamjar, M.J. Munson, C. Thomson, M.M.K. Muqit, I.G. Ganley, mito -QC illuminates mitophagy and mitochondrial architecture in vivo, J. Cell Biol. 214 (2016) 333–345. doi:10.1083/jcb.201603039.

[15] G.F.G. Allen, R. Toth, J. James, I.G. Ganley, Loss of iron triggers PINK1/Parkin-independent mitophagy., EMBO Rep. 14 (2013) 1127–35. doi:10.1038/embor.2013.168.

[16] H. Katayama, T. Kogure, N. Mizushima, T. Yoshimori, A. Miyawaki, A Sensitive and Quantitative Technique for Detecting Autophagic Events Based on Lysosomal Delivery, Chem. Biol. 18 (2011) 1042–1052. doi:10.1016/j.chembiol.2011.05.013.

[17] N. Sun, J. Yun, J. Liu, D. Malide, C. Liu, I.I. Rovira, K.M. Holmström, M.M. Fergusson, Y.H. Yoo, C.A. Combs, T. Finkel, Measuring In Vivo Mitophagy, Mol. Cell. 60 (2015) 685–696. doi:10.1016/j.molcel.2015.10.009.

[18] G. Hernandez, C. Thornton, A. Stotland, D. Lui, J. Sin, J. Ramil, N. Magee, A. Andres, G. Quarato, R.S. Carreira, M.R. Sayen, R. Wolkowicz, R.A. Gottlieb, MitoTimer, Autophagy. 9 (2013) 1852–1861. doi:10.4161/auto.26501.

[19] T.G. McWilliams, A.R. Prescott, B. Villarejo-Zori, G. Ball, P. Boya, I.G. Ganley, A comparative map of macroautophagy and mitophagy in the vertebrate eye, Autophagy. 0 (2019) 1–13. doi:10.1080/15548627.2019.1580509.

[20] A. Terskikh, A. Fradkov, G. Ermakova, A. Zaraisky, P. Tan, A. V Kajava, X. Zhao, S. Lukyanov, M. Matz, S. Kim, I. Weissman, P. Siebert, “Fluorescent timer”: protein that changes color with time., Science. 290 (2000) 1585–8.

doi:10.1126/science.290.5496.1585.

[21] R.C. Laker, J.C. Drake, R.J. Wilson, V.A. Lira, B.M. Lewellen, K.A. Ryall, C.C. Fisher, M. Zhang, J.J. Saucerman, L.J. Goodyear, M. Kundu, Z. Yan, Ampk phosphorylation of Ulk1 is required for targeting of mitochondria to lysosomes in exercise-induced mitophagy, Nat. Commun. 8 (2017). doi:10.1038/s41467-017-00520-9.

[22] M. Lazarou, D.A. Sliter, L.A. Kane, S.A. Sarraf, C. Wang, J.L. Burman, D.P. Sideris, A.I. Fogel, R.J. Youle, The ubiquitin kinase PINK1 recruits autophagy receptors to induce mitophagy, Nature. 524 (2015) 309–314. doi:10.1038/nature14893.

[23] J.M. Gump, A. Thorburn, Sorting cells for basal and induced autophagic flux by quantitative ratiometric flow cytometry, Autophagy. 10 (2014) 1327–1334. doi:10.4161/auto.29394.

[24] M. Mauro-Lizcano, L. Esteban-Martínez, E. Seco, A. Serrano-Puebla, L. Garcia-Ledo, C. Figueiredo-Pereira, H.L.A. Vieira, P. Boya, New method to assess mitophagy flux by flow cytometry, Autophagy. 11 (2015) 833–843. doi:10.1080/15548627.2015.1034403.

[25] H. Zhang, M. Bosch-Marce, L.A. Shimoda, S.T. Yee, H.B. Jin, J.B. Wesley, F.J. Gonzalez, G.L. Semenza, Mitochondrial autophagy is an HIF-1-dependent adaptive metabolic response to hypoxia, J. Biol. Chem. 283 (2008) 10892–10903. doi:10.1074/jbc.M800102200.

[26] T.G. McWilliams, I.G. Ganley, Investigating Mitophagy and Mitochondrial Morphology In Vivo Using mito-QC: A Comprehensive Guide. In: Ktistakis N., Florey O. (eds) Autophagy, in: N. Ktistakis, O. Florey (Eds.), Methods Mol. Biol., Springer New York, New York, NY, 2019: pp. 621–642. doi:10.1007/978-1-4939-8873-0.

[27] J.J. Lee, A. Sanchez-Martinez, A.M. Zarate, C. Benincá, U. Mayor, M.J. Clague, A.J. Whitworth, Basal mitophagy is widespread in Drosophila but minimally affected by loss

of Pink1 or parkin, J. Cell Biol. 217 (2018) 1613–1622. doi:10.1083/jcb.201801044.

[28] T.G. McWilliams, A.R. Prescott, L. Montava-Garriga, G. Ball, F. Singh, E. Barini, M.M.K. Muqit, S.P. Brooks, I.G. Ganley, Basal Mitophagy Occurs Independently of PINK1 in Mouse Tissues of High Metabolic Demand, Cell Metab. 27 (2018) 439-449.e5. doi:10.1016/j.cmet.2017.12.008.

[29] K.C. Dunn, A.E. Aotaki-keen, F.R. Putkey, L.M. Hjelmeland, ARPE-19, A Human Retinal Pigment Epithelial Cell Line with Differentiated Properties, Exp. Eye Res. 62 (1996) 155–170. doi:10.1006/exer.1996.0020.

[30] F. Singh, J. Zoll, U. Duthaler, A.-L. Charles, M. V. Panajatovic, G. Laverny, T.G. McWilliams, D. Metzger, B. Geny, S. Krähenbühl, J. Bouitbir, PGC-1β modulates statin-associated myotoxicity in mice, Arch. Toxicol. (2018). doi:10.1007/s00204-018-2369-7.

[31] E.M. McMillan, J. Quadrilatero, Differential apoptosis-related protein expression , mitochondrial properties , proteolytic enzyme activity , and DNA fragmentation between skeletal muscles, Am J Physiol Regul Integr Comp Physiol. (2011) 531–543. doi:10.1152/ajpregu.00488.2010.

Figure legends:

Figure 1: The mito-QC Counter: a macroinstruction for FIJI to assess mitophagy with the mito-QC reporter. (A) Schematic workflow of the *mito-QC Counter* macro to quantify mitophagy and autophagy assays. (B) Representative widefield fluorescence images showing how the 4 main steps in the *mito-QC Counter* are implemented to quantify mitophagy. Mitophagy was induced in SH-SY5Y cells treated with 1 mM DFP for 24 hours. Scale bar = 10 μm. The pixel intensity graph below depicts how the red dots are quantified based on the intensity profile for the line from A to B. The red discontinued line in STEP4 indicates the mean of red intensity threshold (+0 stdDev), while asterisks indicate peaks of positive mitolysosomes above the red channel threshold.

Figure 2. Assessment of DFP-induced mitophagy in SH-SY5Y and ARPE-19 cells. (A/B) Representative widefield fluorescence images of SH-SY5Y and ARPE-19 cells expressing the *mito*-QC reporter. Mitophagy was stimulated with 1 mM DFP for 24 hours and the number of mitolysosomes was quantified from three independent experiments with >50 cells for experiment. Scale bar = 10 µm. (C) Total number of mitolysosomes counted per cell. (D) Total number of mitolysosomes counted per cell area defined by the manual selection of ROIs. (E) Mitolysosomal area expressed in percent of the cell area. (F) Mean size of the mitolysosomes. Quantification was performed with the following parameters: Radius for smoothing images = 1; Ratio threshold = 0.6; Red channel threshold = Mean + 0 stdDev. Statistics were realised using unpaired Student's t test. n = 3 independent experiments. Statistical significance is displayed as $^{**}p < 0.01$, $^{***}p<0.001$.

Figure 3. Testing mito-QC Counter thresholding settings to assess mitophagy. SH-SY5Y cells expressing the mito-QC reporter were induced with 1 mM DFP for 24 hours and the number of mitolysosomes was quantified either manually or using the *mito-QC Counter* using 40 cells for each treatment (n = 1 experiment). (A) Boxplot assessing the effect of the Red/Green Ratio threshold in *mito-QC Counter*'s quantification. Cells were quantified in batch mode using a Radius for smoothing images = 1, Ratio threshold = 0.6, 0.5, 0.4 and 0.2, and a Red channel threshold = Mean + 0 stdDev. (B) Boxplot assessing the effect of the Radius for smoothing images in *mito-QC Counter*'s quantification. The box that depicts the value for each cell distributed from the 25th to the 75th percentiles and the median value, while the Tukey whiskers depicts the value for each cell distributed 1.5x times the interquartile range (IQR). Cells outside the whiskers range are considered outliers and are depicted as individual dots. Cells were quantified in batch mode using Radius for smoothing images = 0, 1, 2 and 3, Ratio threshold: 0.5, and a Red channel threshold: Mean + 0 stdDev. (C) Boxplot assessing the effect of the stdDev from the mean of mCherry intensity in *mito-QC Counter*'s quantification. Cells were quantified in batch mode using a Radius for

smoothing images = 1, Ratio threshold: 0.5, and Red channel thresholds = Mean + 0, 1, 2, 3, -2 stdDev.

Figure 4: The red-only puncta quantified by the mito-QC Counter are mitolysosomes. Representative confocal images of SH-SY5Y cells expressing the *mito*-QC reporter and stained with anti-LAMP1 antibody. Mitophagy was induced with 1 mM DFP for 24 hours. Zoom insets show representative mitolysosomes (red-only dots) co-localising with LAMP1 structures. White arrows highlight example of quantified mitolysosomes. Scale bar = 10 µm. Quantification was performed with the following parameters: Radius for smoothing images = 2; Ratio threshold = 1.5; Red channel threshold = Mean + 0.5 stdDev.

Figure 5. Assessment of mito-QC Counter's specificity in hypoxia-induced mitophagy with Bafilomycin A1. (A) Representative widefield fluorescence images of ARPE-19 cells expressing the mito-QC reporter and stimulated with hypoxia (1% O_2) for 42 hours and treated with 50 nM Bafilomycin A1 for the last 6 hours of treatment. The number of mitolysosomes was quantified from three independent experiments with >50 cells for experiment. Zoom insets show representative mitolysosomes in both the fluorescence image and mitophagy mask. White arrows highlight example of mitolysosomes. Scale bar = 10 µm. (B) Total number of mitolysosomes counted per cell. (C) Total number of mitolysosomes counted per cell area defined by the manual selection of ROIs. (D) Mitolysosomal area expressed in percent of the cell area. (E) Mean size of the mitolysosomes. Quantification was performed with the following parameters: Radius for smoothing images = 1; Ratio threshold = 0.6; Red channel threshold = Mean + 0 stdDev. Statistics were realised using two-way ANOVA followed by a Bonferroni post-hoc test. n = 3 independent experiments. Statistical significance is displayed as $^{**}p < 0.01$, $^{***}p<0.001$.

Figure 6. The mito-QC Counter assesses in vivo basal mitophagy in skeletal muscle. (A) Representative tile scan of a transverse section of *mito*-QC mouse hindlimb skeletal

muscles. Scale bar = 200 µm. (B) Representative images and zoomed inserts of the Soleus (Sol), the red Gastrocnemius (Red), the mixed Gastrocnemius (Mix), and the white gastrocnemius (Whi), with the (C) corresponding mitophagy channel pictures. Scale bar = 20 µm. (D) Number of mitolysosomes per tissue area. (E) Mitolysosome area expressed in percent of the tissue area. (F) Mean size of the mitolysosomes. (G) Mitochondrial content estimated with the mean intensity per pixel of the *mito*-QC reporter. (H) Number of mitolysosomes per mitochondrial content. (I) Mitolysosome area per mitochondrial content. Quantification was performed with the following parameters: Radius for smoothing images = 1, Ratio threshold = 1, and Red channel threshold = mean + 1 stdDev. Statistics were realised using one-way ANOVAs followed by a Tukey post-hoc test. n = 4 mice. Statistical significance is displayed as *p<0.05, **p<0.01, ***p<0.001, ****p<0.0001.
